# Mesenchymal stem cells alleviate pulmonary fibrosis and gut microbiota dysbiosis in systemic sclerosis

**DOI:** 10.3389/fmicb.2025.1635809

**Published:** 2025-11-26

**Authors:** Biao Ni, Yufang Gong, Bin Li, Lijie Qiu, Kang He, Jintao Guo, Hongkun Fang, Mingjie Gao, Min Chen, Cuie Wei, Weice Sun, Bin Liu, Ming Li, Shaoqiang Wang, Lina Xu

**Affiliations:** 1 Department of Pulmonary and Critical Care Medicine, Center of Respiratory Medicine, Weifang People's Hospital, Shandong Second Medical University, Weifang, Shandong, China; 2 Department of Scientific Research Management, Weifang People's Hospital, Shandong Second Medical University, Weifang, Shandong, China; 3 Department of Rheumatology, Weifang People's Hospital, Shandong Second Medical University, Weifang, Shandong, China; 4 Department of Clinical Medicine, Shandong Second Medical University, Weifang, Shandong, China; 5 Department of Vascular Surgery, Weifang Hospital of Traditional Chinese Medicine, Weifang, Shandong, China; 6 Weifang People's Hospital, Shandong Second Medical University, Weifang, Shandong, China

**Keywords:** mesenchymal stem cells, systemic sclerosis, pulmonary fibrosis, gut microbiota, microbial metabolites

## Abstract

**Introduction:**

Mesenchymal stem cells (MSCs) have shown the potential to alleviate systemic sclerosis (SSc) tissue fibrosis. However, our knowledge of the effects of MSCs on gut microbiota remains limited.

**Methods:**

In this study, we employed a bleomycin induced SSc model to investigate the effects of MSCs on pulmonary fibrosis and gut microbiota in SSc using transcriptomic, microbial metagenomic, and metabolomic analyses.

**Results:**

Our results indicated that MSCs treatment alleviated lung injury in SSc mice. Transcriptomic analysis suggested that the therapeutic effects of MSCs were primarily associated with fatty acid metabolism, PPAR signaling pathway, and AMPK signaling pathway. Furthermore, MSCs restored the relative abundance of microbial taxa, including *Bacteroidota*, *Actinomycetota*, and *Akkermansia muciniphila*, and improved the gut microbiota dysbiosis induced by SSc. Metabolomic data showed that MSCs modulated the dysregulation of trimethyllysine, cholesteryl sulfate expression, and nicotinate and disturbances in nicotinamide metabolism caused by SSc. Correlation analysis demonstrated significant associations among transcriptomic, microbiomic, and metabolomic datasets.

**Discussion:**

Collectively, our findings indicate that MSCs may alleviate SSc pulmonary fibrosis by reshaping the gut microbiota, thereby offering novel scientific insights for the investigation of clinical treatment targets for SSc.

## 1 Introduction

Systemic sclerosis (SSc), commonly referred to scleroderma, is a chronic, inflammatory, and systemic autoimmune disease characterized by extensive microvascular damage, activation of the immune response, and fibrosis of the skin and various internal organ tissues (Volkmann et al., 2023; Distler et al., 2019; Fang et al., 2022). The global incidence of SSc is estimated to range from 8 to 56 cases/million/year, with a high mortality rate and poor prognosis, significantly diminishing patients’ quality of life (Ingegnoli et al., 2018). The etiology and pathogenesis of SSc remain largely unknown, and they are related to many factors, such as heredity and environment. Pulmonary fibrosis is one of the main causes of death in SSc. Currently, there is a lack of effective pharmacological interventions to prevent fibrosis of critical visceral organs and enhance patient survival rate.

The gut microbiota is considered the “second genome” of the host, and both the microbiota and their metabolites are intricately linked to the development, balance, and functionality of the host’s immune system, and they also play a regulatory role in the onset and progression of tissue fibrosis (Belkaid and Hand, 2014; Kawano et al., 2022). The gut microbiota can regulate the organism and immune homeostasis through the “gut–lung axis” and “gut–brain axis” and plays a vital role in maintaining the body’s homeostasis (De Luca and Shoenfeld, 2019; Macpherson et al., 2023). In the context of nonalcoholic steatohepatitis (NASH), the activation of intrahepatic B cells, driven by the gut microbiota, results in liver inflammation and fibrosis via both innate and adaptive immune mechanisms (Barrow et al., 2021). *Bacteroides fragilis* has been shown to mitigate oxidative stress and inflammatory responses by reducing serum lipopolysaccharide (LPS) levels and increasing 1,5-anhydroglucitol (1,5-AG) levels, thereby alleviating renal fibrosis (Zhou et al., 2022). Gut microbial metabolites bile acids play an essential role in inflammation and immune regulation by promoting the generation of regulatory T (Treg) cells (Campbell et al., 2020). Additionally, the gut microbial metabolite 10-hydroxy-cis-12-octaenoic acid has been found to ameliorate hepatic fibrosis by inhibiting TGF-*β*-induced Smad3 phosphorylation and the expression of fibrosis-related genes in hepatic stellate cells (Kasahara N. et al., 2023).

Changes in microbial diversity and activity can influence the resilience of other organisms, thereby impacting their capacity to adapt to changes in physiological conditions. In disease states, the biomass or diversity of gut microbes may either increase or decrease (Shuai et al., 2022; Kasahara K. et al., 2023). Numerous studies have suggested a disease-specific gut microbiota in SSc, characterized by a reduction in commensal genera and an increase in pathobiont genera (Lemos et al., 2022; Andréasson et al., 2022; Volkmann et al., 2017). Studies have demonstrated a significant decrease in the relative abundance of *Bacteroidetes* and an increase in *Firmicutes* in both SSc patients and a bleomycin (BLM)-induced mice model (Tang et al., 2021). Opportunistic pathogenic *Clostridium*, typical oral *Streptococcus*, and homocysteine-producing *Clostridium* are significantly increased in SSc and other fibrosis-prone diseases (Plichta et al., 2021). Arron et al. (2014) reported an increased expression of *Rhodotorula glutinis* sequences in SSc patients, positing that *R. glutinis* may provoke an inflammatory response, thereby contributing to skin fibrosis. These findings suggest that gut microbiota plays a crucial role in the pathogenesis of SSc. Nevertheless, direct investigations into the relationship between gut microbiota and fibrosis in SSc remain lacking.

Mesenchymal stem cells (MSCs) are pluripotent stem cells that can self-renew and secrete antimicrobial peptides, cytokines and other active substances. They exhibit potent immunomodulatory effects and are implicated in processes such as anti-fibrosis, anti-inflammatory responses, anti-tumor activity, promotion of angiogenesis, and neuroprotection (Song et al., 2020; Zhuang et al., 2019). MSCs have been shown to influence the differentiation and function of mononuclear macrophages, T cells, and other immune cells through mechanisms involving direct intercellular contact and paracrine pathways (Galipeau and Sensebe, 2018). Furthermore, MSCs are involved in the regulation of gut microbiota. Evidence suggests that MSCs can significantly restore microbiota diversity and reverse alterations in gut microbiota abundance, thereby modulating dysregulated metabolic pathways in mouse models of colitis (Yang et al., 2022). Additionally, MSCs treatment has been observed to reverse disrupted gut microbiota in mice with hypoxia-induced pulmonary hypertension (PH), indicating that changes in gut microbiota may contribute to the development and progression of PH (Luo et al., 2021). However, it remains unclear whether MSCs modulate the immunoinflammatory response in SSc through interactions with the gut microbiota.

In this study, we hypothesized that MSCs treatment could potentially restore gut microbiota composition and mitigate tissue inflammation in a BLM-induced SSc model. To test this hypothesis, we employed an integrative approach combining microbial metagenomics, non-targeted metabolomics, and transcriptomics to analyze both clinical samples and animal models. Findings of this study may offer novel insights for optimizing biological therapeutic strategies for SSc.

## 2 Materials and methods

### 2.1 Study participants and sample collection

Ten patients aged 40–60 years and age-matched healthy controls, were recruited for this study. Stool and blood samples were collected on-site at Weifang People’s Hospital. The stool samples were initially stored in ice boxes before being transferred into centrifuge tubes. The collection date was documented, and the samples were and stored at −80 °C until further processing. Peripheral blood mononuclear cells (PBMCs) were isolated from the blood samples for RNA extraction and subsequently stored at −80 °C following reverse transcription. The study was approved by the Ethics Committee of Weifang People’s Hospital (approval number: KYLL20241213-6), and informed written consent was obtained from all participants.

### 2.2 Culture and identification of human umbilical cord MSCs (hUC-MSCs)

The human umbilical cord was rinsed with phosphate-buffered saline (PBS) and subsequently sectioned into pieces measuring 1 mm^3^. These tissue blocks were then incubated in DMEM/F12 supplemented with 10% fetal bovine serum (FBS), penicillin (100 U/mL), and streptomycin (100 mg/mL). After approximately 7 days, MSCs emerged from the tissue blocks and proliferated in the surrounding area. Following an additional 3–5 days, the adherent hUC-MSCs were detached using 0.25% trypsin–EDTA. The hUC-MSCs were cultured in 3D FloTrix® mesenchymal stem cell medium (CytoNiche Biotech, Beijing, China) containing penicillin (100 U/mL) and streptomycin (100 mg/mL). The cells were expanded over three to five passages for subsequent use. Flow cytometry analysis was conducted to identify the presence of positive markers CD44 (Biolegend, 338,805), CD73 (Biolegend, 344,003), CD90 (Biolegend, 328,109), and CD105 (Biolegend, 800,505), as well as the absence of negative markers CD34 (Biolegend, 343,603) and CD45 (Biolegend, 304,011) on the hUC-MSCs. The hUC-MSCs were then induced to differentiate into adipocytes (Procell, PD-019), chondrocytes (Procell, PD-018), and osteocytes (Procell, PD-017) using specific stem cell differentiation media (Procell, Wuhan, China), respectively.

### 2.3 Animals and experimental design

The animal experiments were approved by the Animal Ethics Committee of Shandong Second Medical University (approval number: 2022SDL339). Female C57BL/6 mice, aged 6 weeks, were purchased from the Animal Experimental Center of Shandong Second Medical University, and all experiments involving mice were conducted under specific pathogen-free (SPF) conditions. Following a one-week acclimatization period, mice in the model group received subcutaneous injections of BLM (100 μL, 0.5 mg/mL) on the back daily for 4 weeks. The healthy control group (CON) was administered an equivalent volume of PBS. Subsequently, the model mouse was randomly allocated into two groups: the hUC-MSC treatment group, receiving 5 × 10^5^ hUC-MSCs per mouse (MSC), and the disease control group, receiving 100 μL PBS per mouse (SSC). Caudal intravenous therapy was administered during the fifth and seventh weeks. Upon completion of the treatment period (the eighth week), all mice were sacrificed and lung tissues and stool samples were collected for further analysis.

### 2.4 Histopathologic and pathology assessment

A portion of the lung tissues from the mice was fixed in 4% paraformaldehyde, while the remaining tissues were preserved at −80 °C. Subsequent to dehydration and paraffin embedding, the lung tissues were sectioned using a rotary microtome to a thickness of 5 μm (He et al., 2024). The sections were stained with hematoxylin and eosin (H&E) as well as Masson’s trichrome stain. The expression of *α*-SMA was assessed by a primary antibody against α-SMA (dilution 1:500, CST, 19245 T) incubated overnight at 4 °C, followed by a horseradish peroxidase (HRP)-labeled goat anti-rabbit secondary antibody (dilution 1:200, Servicebio, GB23303) for 50 min at 25 °C. Similarly, COL1A1 expression was assessed using a primary antibody COL1A1 (dilution 1:100, Affinity, AF7001) overnight at 4 °C and HRP-labeled goat anti-rabbit secondary antibody (dilution 1:200, Servicebio, GB23303) for 50 min at 25 °C. The samples were digitally scanned using the application system EasyScan 6 system (Motic, Xiamen, China) to obtain high-resolution images.

### 2.5 Quantitative real-time PCR (qPCR)

Human blood PBMCs and mice lung tissues were homogenized using TriQuick Reagent (Solarbio, Beijing, China) to facilitate the extraction of total RNA, employing the FastPure Cell/Tissue Total RNA Isolation Kit V2 (Vazyme, Nanjing, China). Complementary DNA (cDNA) was synthesized from the total RNA samples by reverse transcription via HiScript II Q-RT SuperMix for quantitative PCR (qPCR) (Vazyme, Nanjing, China). The qPCR assays were performed using Taq Pro Universal SYBR qPCR Master Mix (Vazyme, Nanjing, China) on the Applied Biosystems QuantStudio 5 real-time PCR system (Thermofisher, USA). The sequences of the primers used in the assays are detailed in Supplementary Table S1.

### 2.6 Microbial metagenomics analysis

The total genomic DNA of stool samples was extracted using the Magnetic Soil and Stool DNA Kit (TianGen, Beijing, China) following the manufacturer’s instructions. The quality and quantity of DNA were verified with NanoDrop and agarose gel. The genomic DNA was randomly sheared into short fragments, and sequencing libraries were generated. The obtained fragments were end-repaired, A-tailed, and further ligated using an Illumina adapter. The fragments with adapters were amplified by PCR, and the size was selected and purified. The library was checked with Qubit, qPCR was used for quantification, and a bioanalyzer was used for size distribution detection. Quantified libraries were pooled and sequenced on the Illumina platform according to the required effective library concentration and data amount. Readfq^1^ was used to preprocess raw data from the Illumina PE150 sequencing platform (Novogene, Beijing, China) to obtain clean data for subsequent analysis. MEGAHIT software was used for assembly analysis of clean data, as previously described (Karlsson et al., 2013; Nielsen et al., 2014). DIAMOND software^2^ (Buchfink et al., 2015) was used for the alignment of Unigene sequences with those of bacteria, fungi, archaea, and viruses extracted from NCBI’s NR database.^3^ LCA algorithm (applied to systematic taxonomy of MEGAN software^4^ was adopted to determine the species annotation information of the sequence; Huson et al., 2011). Based on the results of LCA annotation, the abundance of each sample at each taxonomy (kingdom, phylum, class, order, family, genus, or species) was acquired. Alpha diversity indices included several observed species, Shannon, Chao1, and ACE. Beta diversity analysis was used to evaluate differences among different groups. Group differences were statistically tested using Analysis of Similarities (ANOSIM) via the R vegan package. Linear discriminant analysis effect size (LEfSe) was performed to investigate potential indicator species using the Kruskal–Wallis (KW) sum-rank test within gut microbes enriched explicitly in a specific group (*p* < 0.05, LDA ≥ 4) (Xiang et al., 2020; Zhao et al., 2019; Segata et al., 2011). Raw sequences data of microbial metagenomics are available in the NCBI Sequence Read Archive (SRA) database under accession number PRJN1124507.^5^

### 2.7 Stool metabolomics analysis

Stool samples from humans (*n* = 10 per group) and mice (*n* = 6 per group) were subjected to non-targeted metabolomics analysis. Each sample (100 mg) was individually using liquid nitrogen, and the homogenate was resuspended with prechilled 80% methanol and vortexed well. The samples were then incubated on ice for 5 min and centrifuged at 15,000 × g at 4 °C for 20 min. A portion of the supernatant was diluted to achieve a final concentration containing of 53% methanol using Liquid Chromatograph Mass Spectrometer (LC–MS) grade water, and then centrifuged at 15000 × g at 4 °C for 20 min. Finally, the supernatant was injected into a liquid chromatography-tandem mass spectrometer (LC–MS/MS) system for analysis (Want et al., 2013). Ultra High Performance Liquid Chromatography–Tandem Mass Spectrometry (UHPLC–MS/MS) analyses were performed using a Vanquish UHPLC system (ThermoFisher, Germany) coupled with an Orbitrap Q ExactiveTM HF-X mass spectrometer (Thermo Fisher, Germany) at Novogene Co., Ltd. (Beijing, China). Principal components analysis (PCA) and partial least squares discriminant analysis (PLS–DA) were performed at metaX (Wen et al., 2017). The metabolites with VIP > 1, *p* < 0.05, and fold change (FC) ≥ 1.5 or ≤ 0.667 were considered differential metabolites. The functions of these metabolites and metabolic pathways were studied using the Kyoto Encyclopedia of Genes and Genomes (KEGG) database.

### 2.8 Transcriptome profiling

The lung tissues from six mice per group were processed for transcriptomic analysis. The integrity of the RNA was evaluated utilizing the RNA Nano 6,000 Assay Kit of the Bioanalyzer 2,100 system (Agilent Technologies, CA, USA). The library preparation workflow included mRNA enrichment, cDNA generation, and end repair to generate blunt ends, adaptor ligation, and PCR amplification. Subsequently, PCR products were purified, and the library quality was assessed using the Agilent Bioanalyzer 2,100 system. Sequencing was performed on an Illumina Novaseq platform (Novogene, Beijing, China), and 150-bp paired-end reads were generated. Clean data (clean reads) were obtained by removing reads containing the adapter, reads containing ploy-N, and low-quality reads from raw data (raw reads). All the downstream analyses were based on these clean, high-quality data. Differentially expressed genes (DEGs) were identified based on a fold change (FC) ≥ 1.5 and *p* < 0.05. Gene Ontology (GO) and KEGG pathway enrichment analysis of DEGs was implemented using the clusterProfiler R package, in which gene length bias was corrected. GO terms with corrected *p* < 0.05 were considered significantly enriched by DEGs.

### 2.9 Statistical analysis

Gut microbiota, metabolomics, and RNA-seq analysis were analyzed and visualized using R software (version 4.3.3, http://www.r-project.org). Principal coordinate analysis (PCoA; packages: vegan, ggplot2) based on the Bray–Curtis distance matrix was performed to compare the differences in microbial community structure. Permutational Multivariate Analysis of Variance (PERMANOVA) (package: vegan, adonis function) based on the Bray–Curtis distance matrix was employed to assess the significant differences in the gut microbiota (Ni et al., 2022; Shuai et al., 2022). Mantel tests (package: corrplot, ggplot2) and Spearman correlation analysis (package: vegan, ggcor, ggplot2) were performed to observe the correlation between the extracellular matrix, collagen-related genes, differential taxa, and metabolites. All statistical differences in this study were considered significant at *p* < 0.05.

## 3 Results

### 3.1 Gut microbiota dysbiosis in patients with SSc may be associated with SSc inflammation

**Figure 1:**
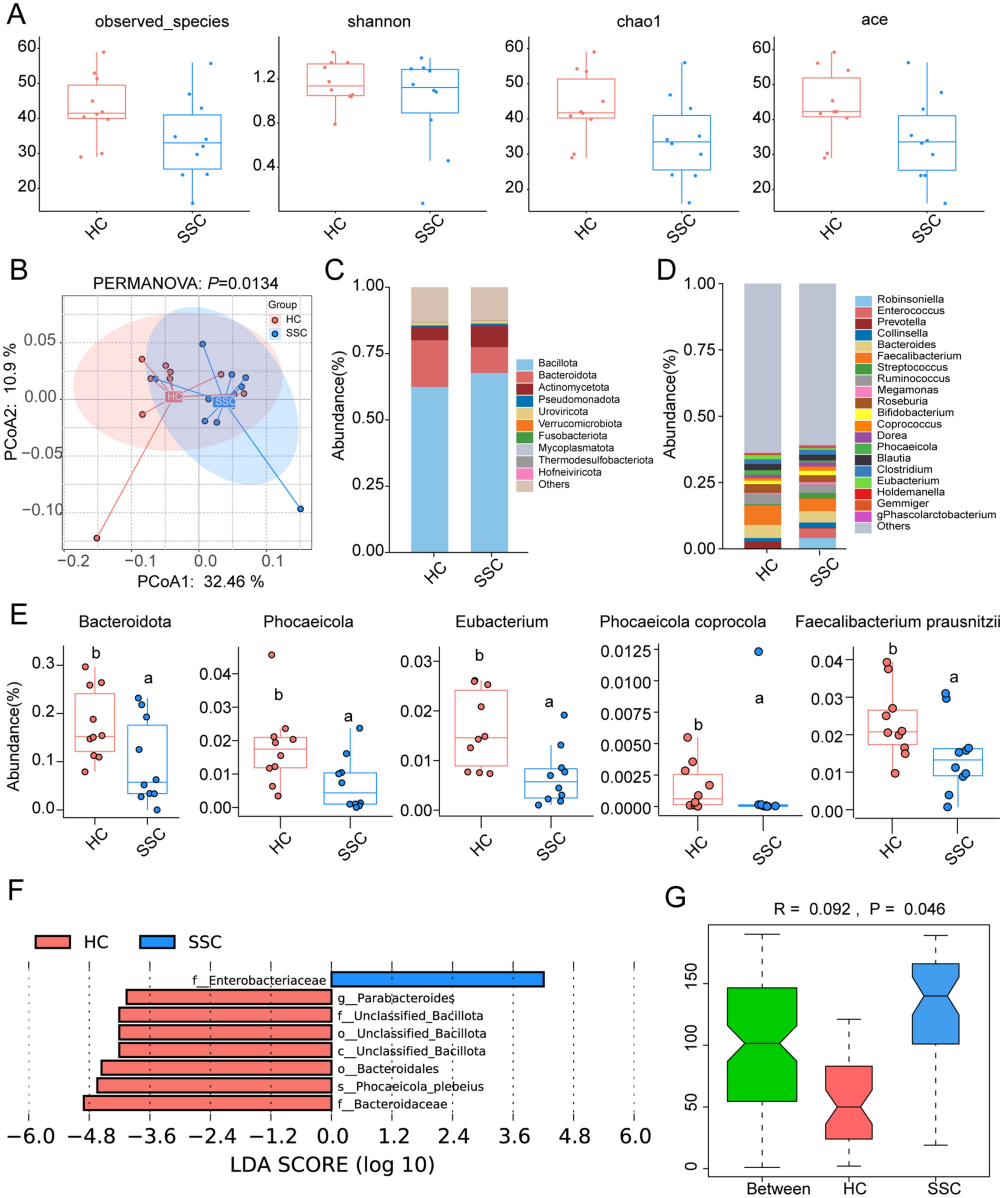
Gut microbiota dysbiosis in patients with SSc. **(A)** Alpha diversity of gut microbiota in healthy controls (HC) and patients with SSc (SSC). **(B)** Principal coordinate analysis (PCoA) of microbiota community based on Bray-Curtis distance at species level. Relative abundance of gut microbiota at the phylum **(C)** and genus **(D)** level. **(E)** Relative abundance of the significantly altered microbial taxa from the two groups. **(F)** LDA (Linear discriminant analysis) discriminatory results of LEfSe analysis. Only taxa with LDA scores of more than 4 were presented. **(G)** ANOSIM analysis of gut microbiota biological function between the HC and SSC groups.

The diversity and structural composition of microbial communities were analyzed based on the results of microbial metagenome sequencing. Compared with healthy controls (HC), patients with SSc (SSC) exhibited a reduction in the *α*-diversity indices (observed species, Shannon, Chao1, ACE) of the gut microbial community (Figure 1A). Principal coordinate analysis (PCoA) at the species level demonstrated a significant separation between gut microbial communities of the HC and SSC groups (PERMANOVA: *p* = 0.0134, Figure 1B). Consistent findings were observed through ANOSIM and cluster analysis (*p* = 0.046, Supplementary Figures S1A,B), suggesting potential dysbiosis in the gut microbial community of SSc patients. *Bacillota* (65.01%), *Bacteroidota* (13.69%), and *Actinomycetota* (6.53%) were the main microbial phylum. Compared with the HC group, the relative abundance of *Bacteroidota*, *Phocaeicola*, *Eubacterium*, *Phocaeicola coprocola*, *Faecalibacterium prausnitzii*, and *Parabacteroides* in the SSC group were significantly decreased (Figures 1C–E; Supplementary Figures S1C,D). By contrast, the relative abundances of *Sphingomonadales*, *Myxococcales*, *Sphingomonadaceae*, and *Klebsiella* were significantly increased (Supplementary Figure S1D). Linear discriminant analysis effect size (LEfSe) results showed that *Parabacteroides*, *Bacteroidales*, *Bacteroidaceae*, and *Phocaeicola plebeius* were significantly enriched in the HC group, while *Enterobacteriaceae* was significantly enriched in the SSC group (Figure 1F). ANOSIM based on functional abundance at the KEGG KO level revealed a significant differentiation in gut microbial function between the HC and SSC groups (*R* = 0.092, *p* = 0.046, Figure 1G). Compared to the HC group, carbohydrate metabolism, cellular community, amino acid metabolism, nucleotide metabolism, lipid metabolism, metabolism of cofactors and vitamins were predicted to be associated with alterations of gut microbiota in SSc patients (Supplementary Figure S2). Collectively, these findings suggest that gut microbiota dysbiosis is present in SSc, potentially influencing the progression of the disease through the aforementioned pathways.

qPCR analysis revealed a significant upregulation in the expression levels of TNF-α and IL-6 in the blood of patients with SSc (*p* < 0.05, Supplementary Figure S3A), suggesting an enhancement of systemic inflammation in these patients. Correlation analysis further demonstrated that the expression levels of inflammatory cytokines TNF-α and IL-6 were significantly associated with the relative abundance of microbial taxa, including S*phingomonadales*, *Myxococcales*, *Sphingomonadaceae*, and *Klebsiella* (*p* < 0.05, Supplementary Figure S3B). Based on these findings, we propose the hypothesis that dysbiosis of the gut microbiota may play a role in the immune and inflammatory responses observed in SSc and could be implicated in its pathogenesis.

### 3.2 Dysregulated gut microbial metabolism in SSc patients

**Figure 2:**
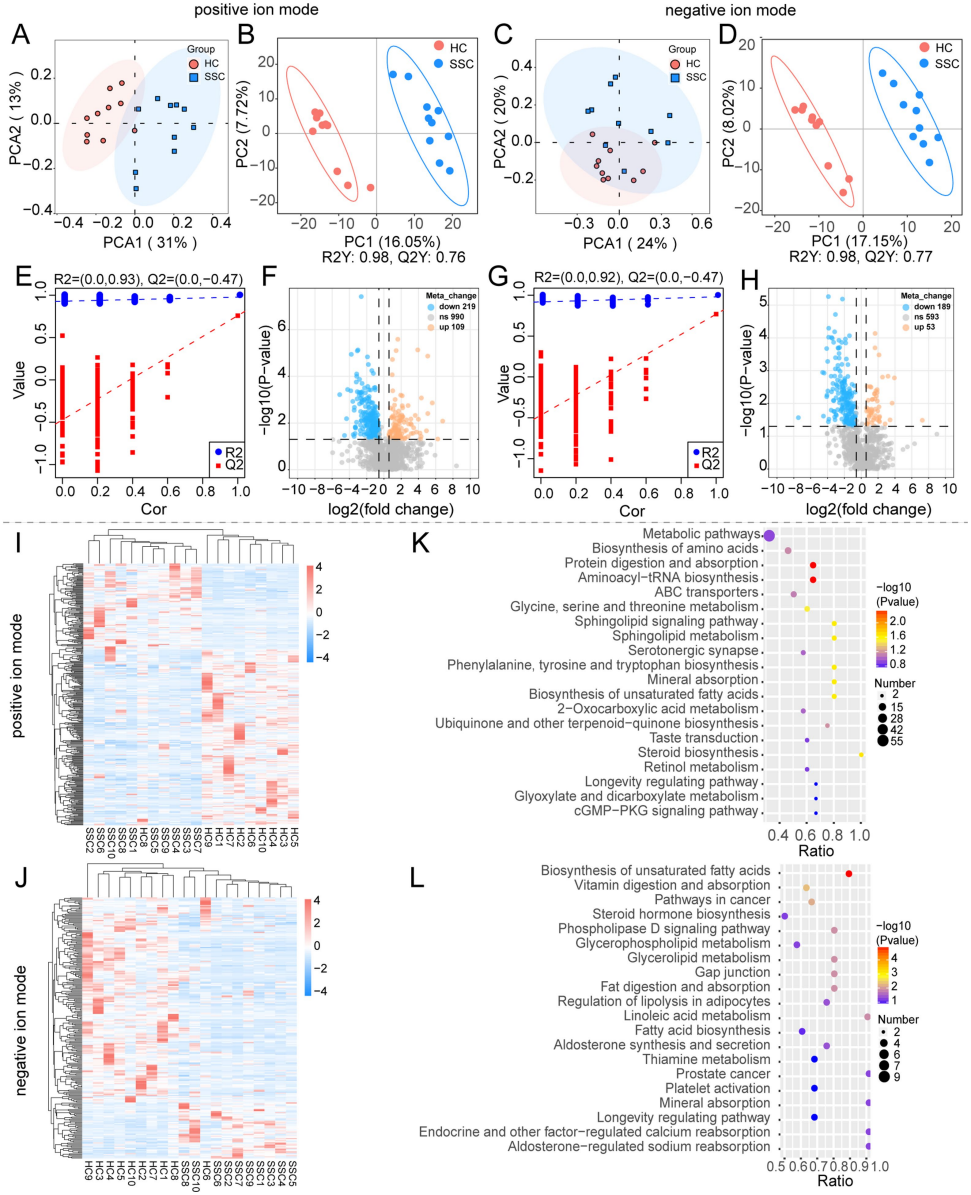
Gut microbial metabolism is dysregulated in SSc patients. **(A,C)** Principal component (PCA) analysis of gut microbial metabolites in healthy controls (HC) and SSc patients (SSC) in positive and negative ion modes. Scatter plot **(B,D)** and rank validation plot **(E,G)** of gut microbial metabolites in HC and SSC group by partial least squares discriminant analysis (PLS-DA). **(F,H)** Volcano plot of differential metabolites in HC and SSC group. **(I,J)** Heatmap of differential metabolites of intestinal microorganisms between HC and SSC group. **(K,L)** Bubble plot of KEGG enrichment analysis of differential metabolites of intestinal microorganisms between HC and SSC group.

To understand the differences in gut microbial metabolic function, we performed an untargeted metabolomics analysis of the collected stool samples. Principal Component Analysis (PCA) and Partial Least Squares Discriminant Analysis (PLS-DA) revealed a significant separation in the gut microbiota metabolite composition between the HC and SSC groups (Figures 2A–E,G), suggesting altered patterns in the composition of gut microbial metabolites in SSc patients. Volcano plot analysis was performed to identify differentially expressed gut microbiota metabolites (*p* < 0.05, FC = 1.5). In total, 1,318 and 835 metabolites were detected in positive and negative ion modes, respectively. Within the positive ion mode, 219 metabolites were significantly down-regulated, while 109 were significantly up-regulated in the stool samples of SSc patients (Figure 2F). In the negative ion mode, 189 metabolites were significantly down-regulated, and 53 metabolites were significantly up-regulated in the feces of SSc patients (Figure 2H). A heatmap analysis illustrated distinct patterns of differentially expressed metabolites (DEMs) accumulation within each group, with a clear distinction between the HC and SSC groups (Figures 2I,J). Subsequently, we conducted a KEGG enrichment analysis of the differential metabolites. The differentially expressed metabolites were mainly involved in the protein digestion and absorption, aminoacyl-tRNA biosynthesis, and biosynthesis of unsaturated fatty acids (Figures 2K,L). These data reveal that gut microbial metabolism in SSc patients were dysregulated.

### 3.3 Identification of hUC-MSCs

**Figure 3:**
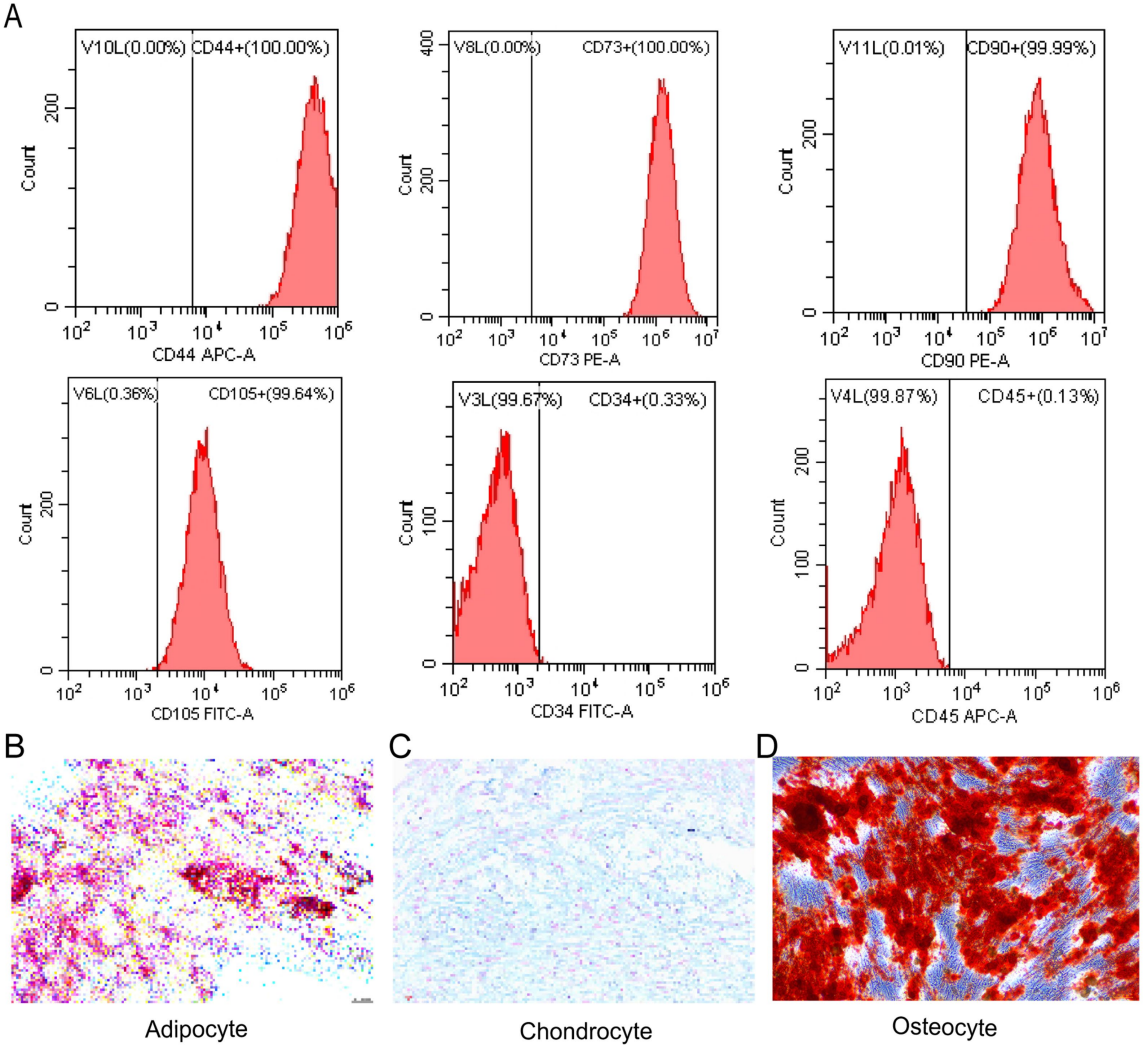
Identification of hUC-MSCs. **(A)** Flow cytometry plots showing the surface markers of the hUC-MSCs. **(B)** Oil Red O staining of hUC-MSCs underwent adipogenic differentiation for 16 days. **(C)** Alcian blue staining of hUC-MSCs underwent chondrogenic induction for 21 days. **(D)** Alizarin red staining of hUC-MSCs underwent osteogenic differentiation for 18 days.

As previously outlined, hUC-MSCs were further characterized in accordance with the criteria established by the International Society of Cellular Therapy (Dominici et al., 2006). After approximately 2 days in culture, the MSCs adhered to the culture flask, exhibiting a spindle-shaped morphology. The surface markers of the hUC-MSCs were detected by flow cytometry, revealing high expression levels of CD44 (100.00%), CD73 (100.00%), CD90 (99.99%), and CD105 (99.64%) were highly expressed in hUC-MSCs, while CD45 (0.33%) and CD34 (0.13%) were minimally expressed (Figure 3A). Following adipogenic, chondrogenic, and osteogenic inductions, the hUC-MSCs showed strong capacities of differentiation into adipocytes, chondrocytes, and osteocytes, respectively, as evidenced by Oil Red O staining (Figure 3B), Alcian Blue staining (Figure 3C), and Alizarin Red staining (Figure 3D).

### 3.4 MSCs alleviated tissue damages in bleomycin-induced SSc mice

**Figure 4:**
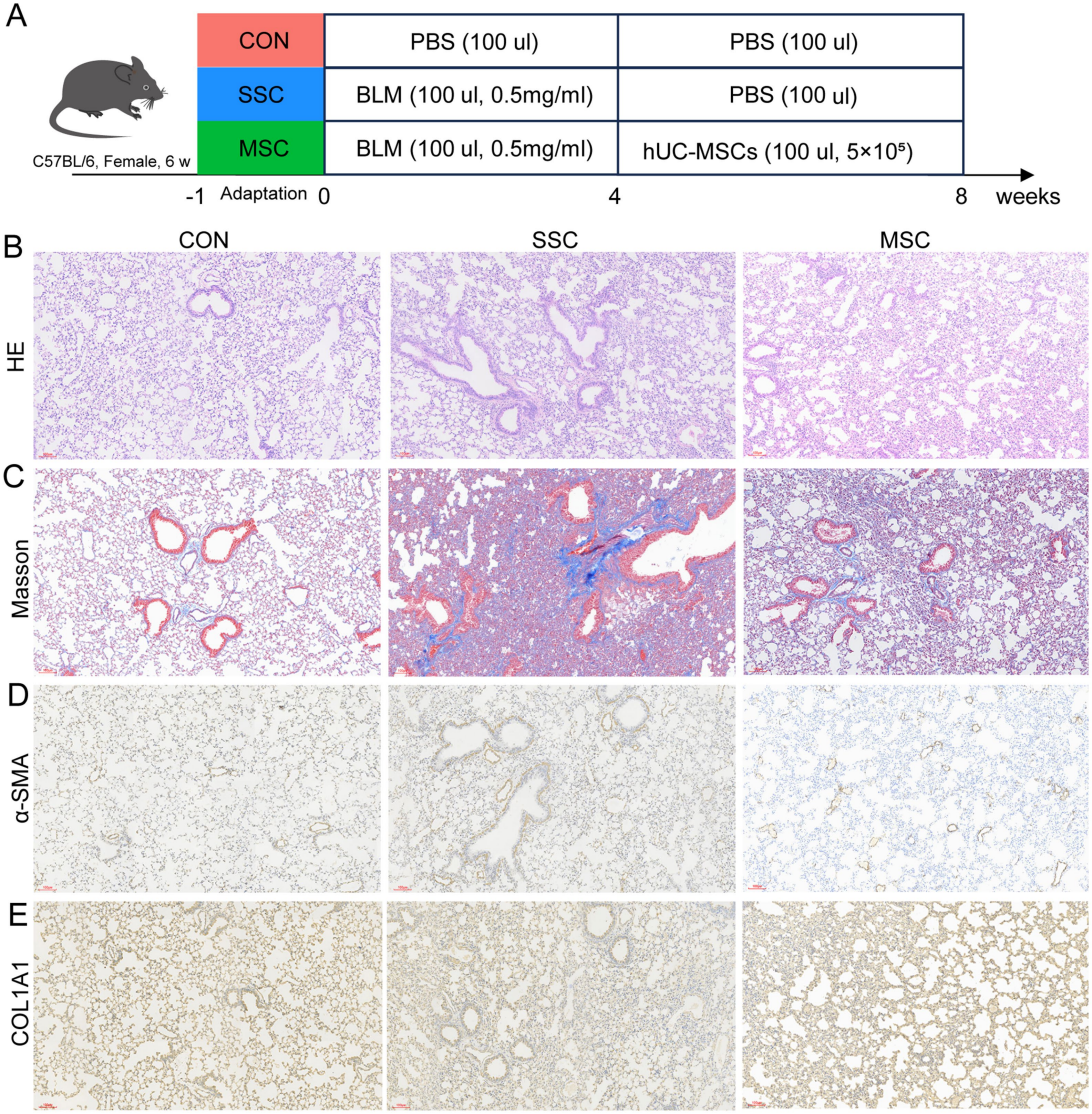
MSCs alleviated tissue damages in bleomycin-induced SSc mice. **(A)** Design of SSC model in mice induced by bleomycin. H&E **(B)** and Masson **(C)** staining of representative sections of mice lung tissue. Expression of *α*-SMA **(D)** and COL1A1 **(E)** in lung tissue of mice.

Following a one-week acclimatization period, the C57BL/6 mice were randomly divided into three groups (*n* = 6 per group): healthy control (CON), bleomycin treatment (SSC), and MSCs therapy (MSC) groups (Figure 4A). Lung tissues from the mice were subsequently collected for pathological staining analysis. Compared to the CON group, H&E and Masson staining revealed a significant increase in inflammatory cell infiltration and collagen deposition in the lung tissues of the SSC group, whereas these symptoms were markedly reduced following MSCs treatment (Figures 4B,C). Immunohistochemistry (IHC) results indicated an upregulation of fibrosis-related proteins *α*-SMA and COL1A1 in the lung tissues of the SSC group, which was subsequently diminished in the MSC group (Figures 4D,E). These results suggest that MSCs therapy may mitigate pulmonary inflammatory injury and fibrosis in SSc mice.

### 3.5 Effects of MSCs treatment on the lung transcriptome

**Figure 5:**
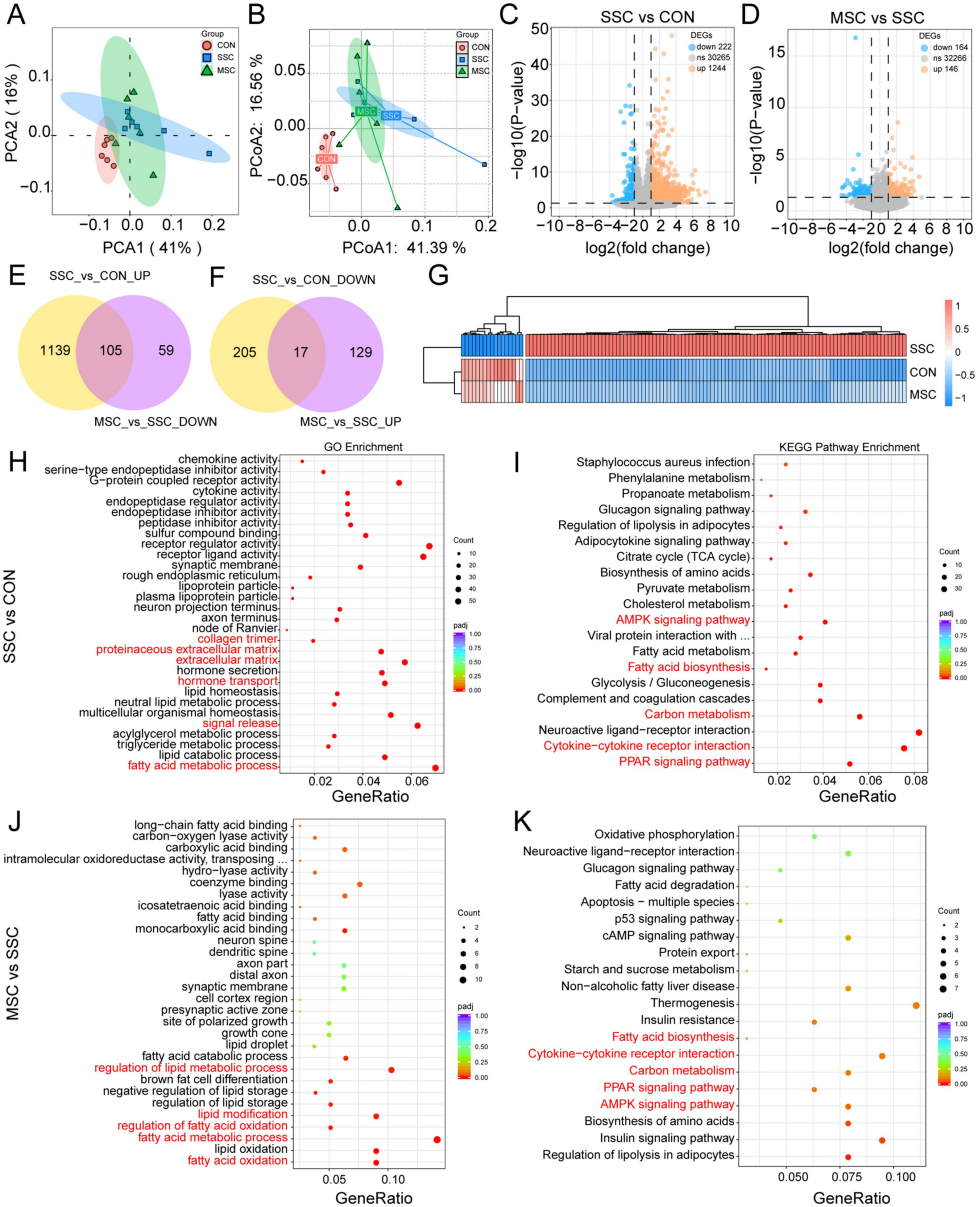
Effects of MSCs treatment on the lung transcriptome. **(A)** Principal component (PCA) and **(B)** Principal coordinate analysis (PCoA) analysis of the pattern of lung tissue gene transcript in mice. Volcano plot of DEGs in SSC vs. CON **(C)** and MSC vs. SSC **(D)**. **(E,F)** The Venn diagram shows the overlap of DEGs between different comparison combinations. **(G)** Heatmap of DEGs that were significantly up-regulated in SSC and significantly down-regulated after MSCs treatment. Bubble plot of GO enrichment analysis of DEGs in SSC vs. CON **(H)** and MSC vs. SSC **(J)**. Bubble plot of KEGG enrichment analysis of DEGs in SSC vs. CON **(I)** and MSC vs. SSC **(K)**.

To further investigate the molecular mechanisms underlying MSCs therapy in SSc-induced pulmonary fibrosis, transcriptome sequencing was performed in CON, SSc, and MSC -treated mice. PCA and PCoA demonstrated a significant divergence in gene expression patterns between the CON and SSC groups (Figures 5A,B), whereas the MSC-treated group exhibited gene expression profiles more closely aligned with the CON group. This observation suggests that MSC treatment may modulate gene expression in SSc mice. Volcano plot analysis was used to find differentially expressed genes (DEGs) (*p* < 0.05, FC = 2). Compared with the CON mice, 222 genes were significantly down-regulated and 1,244 genes were significantly up-regulated in the SSc mice (Figure 5C). Compared with the SSC mice, 146 genes were significantly down-regulated and 164 genes were significantly up-regulated after MSCs treatment (Figure 5D). Further analysis revealed that MSC treatment reversed the expression of specific genes in the SSC group, with 105 genes initially up-regulated becoming down-regulated, and 17 genes initially down-regulated becoming up-regulated (Figures 5E–G).

GO and KEGG analyses were performed to delineate the significant functional of DEGs induced by SSC and MSCs treatment. GO enrichment analysis showed that genes were significantly up-regulated in the SSC group were primarily associated with the fatty acid metabolic process, signal release, hormone transport, and cellular components, including the extracellular matrix, proteinaceous extracellular matrix, and collagen trimer (Figure 5H). Conversely, genes were significantly down-regulated following MSCs treatment were mainly enriched in the fatty acid metabolic process, regulation of lipid metabolic process, fatty acid oxidation, lipid modification, and regulation of fatty acid oxidation (Figure 5J). The KEGG pathway enrichment analysis indicated that pathways such as the AMPK signaling pathway, PPAR signaling pathway, fatty acid biosynthesis, carbon metabolism, and cytokine−cytokine receptor interaction were significantly up-regulated in the SSC group, but were significantly down-regulated following MSC treatment (Figures 5I,K). These findings suggest that MSCs may alleviate pulmonary fibrosis in SSC mice by regulating these pathways.

### 3.6 MSCs improved the gut microbiota dysbiosis in SSc mice

**Figure 6:**
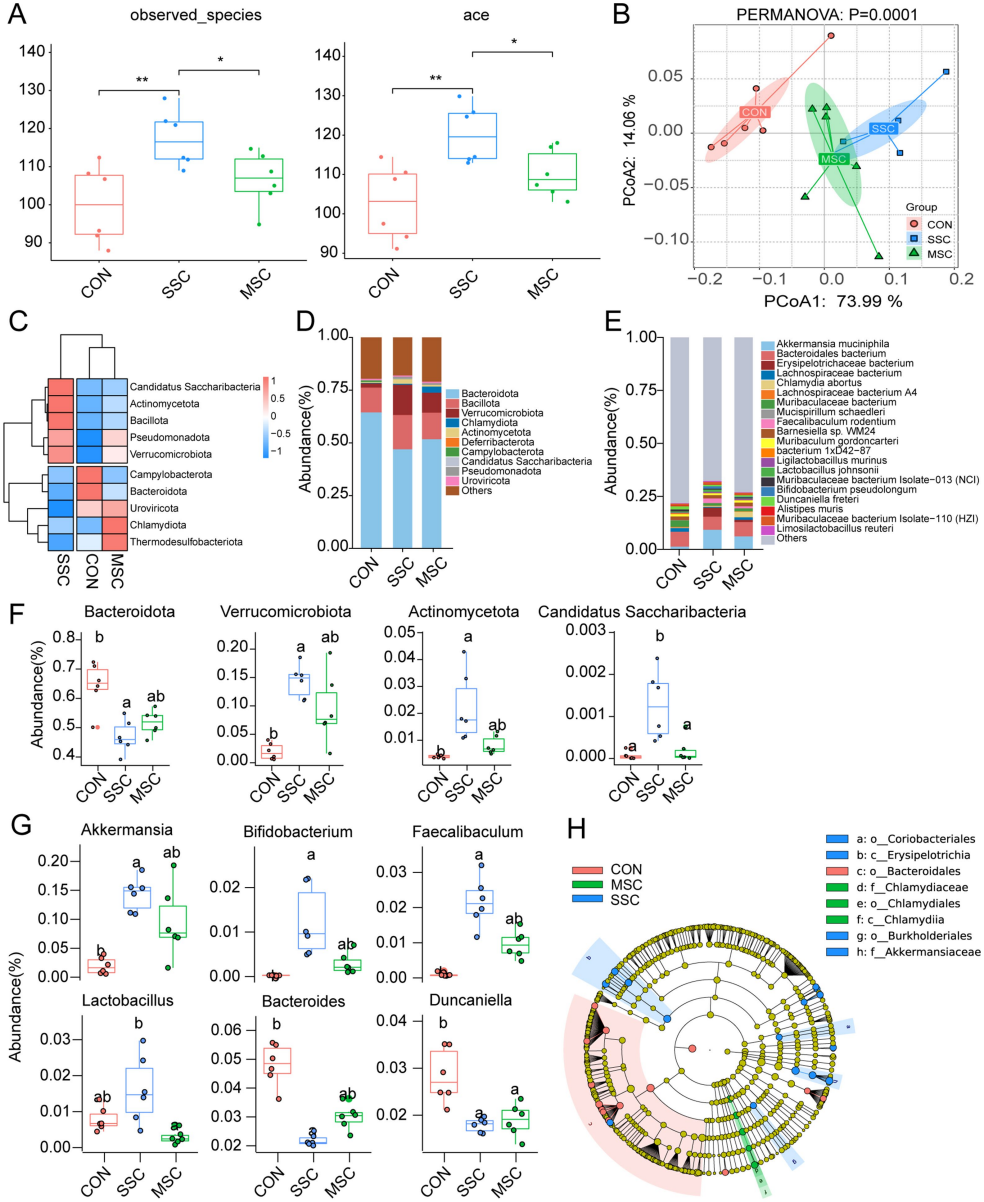
MSCs improved the gut microbiota dysbiosis in SSc mice**. (A)** Alpha diversity of gut microbiota in mice. **(B)** Principal coordinate analysis (PCoA) of microbiota community based on Bray-Curtis distance at phylum level. **(C)** Clustering heatmap of gut microbiota at the phylum level. Relative abundance of gut microbiota at the phylum **(D)** and genus **(E)** level. Relative abundance of the significantly altered microbial taxa at the phylum **(F)** and genus **(G)** level from the three groups. **(H)** LDA (Linear discriminant analysis) discriminatory results of LEfSe analysis. Only taxa with LDA scores of more than 4 were presented.

Compared with the CON mice, the *α*-diversity indices (observed species, Shannon, Chao1, and ACE) of the gut microbial community were significantly higher in SSc mice (SSC), while the administrations of MSCs could reduce these changes (Figure 6A; Supplementary Figure S4A). PCoA at both the phylum and species levels demonstrated a significant separation of the gut microbial communities in the SSC group. The microbial communities of the CON and SSC groups were significantly separated, whereas the microbial communities in the MSC group exhibited a divergence from the SSc group, showing a greater similarity to the CON group (PERMANOVA: *p* = 0.0001, Figure 6B; Supplementary Figure S4B). Consistent findings were observed in the group-paired PCoA analysis (*p* < 0.05, Supplementary Figures S4C–E). Clustering analysis further revealed that the microbial communities of the CON and MSC groups clustered into a single large branch, whereas the SSC group formed a separate branch (Figure 6C; Supplementary Figure S4F).

Similar to the human gut microbiota, *Bacteroidota* (54.35%) and *Bacillota* (13.54%) were the main microbial phylum in mice. At the phylum level, compared with the CON group, the relative abundances of *Bacteroidota* in the SSC group were significantly decreased, while that of *Verrucomicrobiota*, *Actinomycetota*, and *Candidatus Saccharibacteria* were significantly increased, and then increased or decreased after MSCs treatment (Figures 6D,F). At the genus level, compared with CON group, the relative abundance of *Bacteroidesin* and *Duncaniella* in the SSC group was significantly decreased, while that of *Akkermansia*, *Bifidobacterium*, *Faecalibaculum*, and *Lactobacillus* in SSC group was significantly increased, and then increased or decreased after MSCs treatment (Figures 6E,G). At the species, compared with CON mice, the relative abundance of *Akkermansia muciniphila*, *Bifidobacterium pseudolongum*, *Erysipelotrichaceae bacterium*, *Lactobacillus johnsonii*, *Muribaculaceae bacterium* Isolate-110 (HZI), and *Faecalibaculum rodentium* increased in SSc mice, then decreased with MSCs treated (Supplementary Figures S4G,H). Linear discriminant analysis effect size (LEfSe) results showed that *Bacteroidales*, *Bacteroides*, *Helicobacter ganmani*, *Duncaniella freteri*, *Muribaculaceae bacterium*, *bacterium_1xD42 87*, *Bacteroides_sp_CAG_927*, and *Bacteroides acidifaciens* were significantly enriched in the CON group. *Erysipelotrichia*, *Coriobacteriales*, *Burkholderiales*, *Akkermansiaceae*, *Akkermansia*, *Faecalibaculum*, *Lactobacillus*, *Lactobacillus_johnsonii*, *Faecalibaculum rodentium*, *Ligilactobacillus murinus*, *Bifidobacterium pseudolongum*, *Erysipelotrichaceae bacterium*, *Limosilactobacillus reuteri*, and *Akkermansia muciniphila* were significantly enriched in the SSC group. *Chlamydiia*, *Chlamydiales*, *Chlamydiaceae*, *Chlamydia*, and *Chlamydia abortus* were significantly enriched in the MSC group (Figure 6H). Overall, these results had demonstrated that the structure of gut microbiota in SSc mice was significantly changed compared with that in the control mice, meaning that the gut microbiota of SSc mice is imbalanced. These results are consistent with those of patients with SSc. MSCs therapy could effectively affect the diversity and composition of gut microbiota in SSc mice. As a result, the administrations of MSCs help to alleviate gut microbiota dysbiosis in SSc mice.

### 3.7 MSCs improved the gut microbial metabolism dysregulate in SSc mice

**Figure 7:**
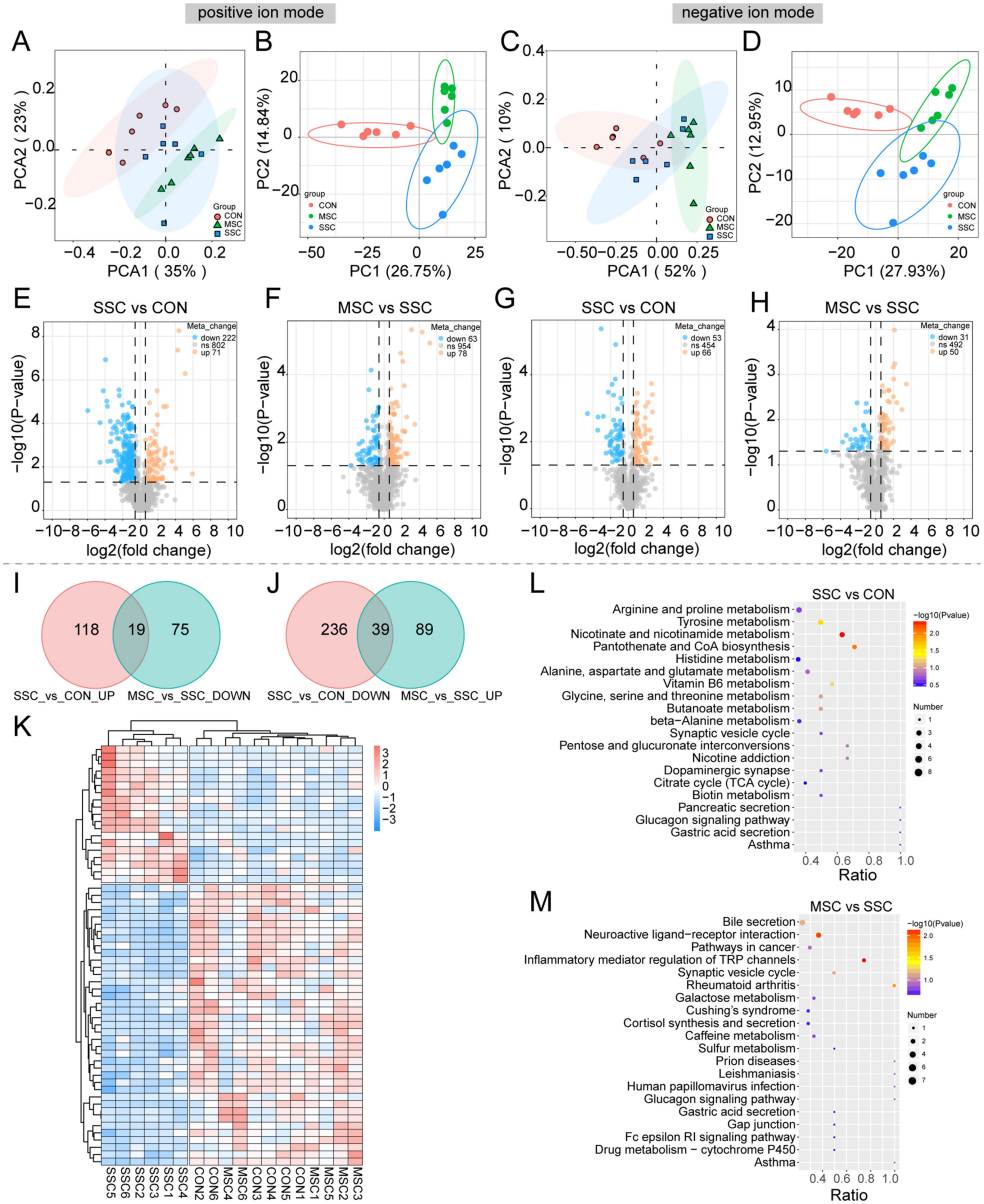
MSCs improved the gut microbial metabolism dysregulate in SSc mice. Principal component (PCA) analysis of gut microbial metabolites in mice in positive **(A)** and negative **(C)** ion modes. PLS-DA analysis of gut microbial metabolites in mice in positive **(B)** and negative **(D)** ion modes. **(E–H)** Volcano plot of differential metabolites in SSC vs. CON, MSC vs. SSC. The Venn diagram shows the overlap of DEMs in SSC vs. CON **(I)**, MSC vs. SSC **(J)**. **(K)** Heatmap of DEMs among the three mice groups. Bubble plot of KEGG enrichment analysis of DEMs between CON vs. SSC group **(L)**, SSC vs. MSC group **(M)**.

To investigate metabolite changes induced by gut microbiota after MSCs treatment, we detected non-targeted metabolites by UHPLC–MS/MS. Similar to the results in human samples, PCA and PLS–DA analysis showed that the gut microbiota metabolite composition between the CON and SSC groups was significantly separated (Figures 7A–D), indicates that the gut microbial metabolism dysregulated in SSc mice. The gut microbiota metabolite composition of MSC group were more similar with CON group, this implies that MSCs treatment may help to altered the dysregulated of gut microbial metabolites in SSc mice. 1,095 and 573 metabolites were detected in the positive and the negative ion modes, respectively. Compared to the CON group, BLM treatment resulted in significant alterations in metabolite levels, with 137 (positive: 71, negative:66) metabolites up-regulated and 275 (positive: 222, negative: 53) metabolites down-regulated (Figures 7E,G). MSCs treatment increased the levels of 128 (positive: 78, negative: 50) metabolites and decreased the levels of 94 (positive: 63, negative:31) metabolites (Figures 7F,H). To refine the list of DEMs, a Venn analysis was conducted across the three groups. MSCs treatment significantly altered the expression trends of 58 metabolites, which were significantly up-regulated or down-regulated in the SSC group (Figures 7I,J). The heatmap analysis demonstrated that the microbial metabolites from the CON and MSC groups clustered into a single large branch, whereas the SSC group formed a distinct branch (Figure 7K). KEGG enrichment analysis results showed that the DEMs were mainly involved in nicotinate and nicotinamide metabolism, pantothenate and CoA biosynthesis, tyrosine metabolism, neuroactive ligand−receptor interaction, inflammatory mediator regulation of TRP channels (Figures 7L,M). These data indicated that MSCs treatment could alleviate metabolic disorders by altering the gut microbial metabolic process in SSc.

### 3.8 MSCs regulates tissue fibrosis by affecting gut microbiota and metabolites

**Figure 8:**
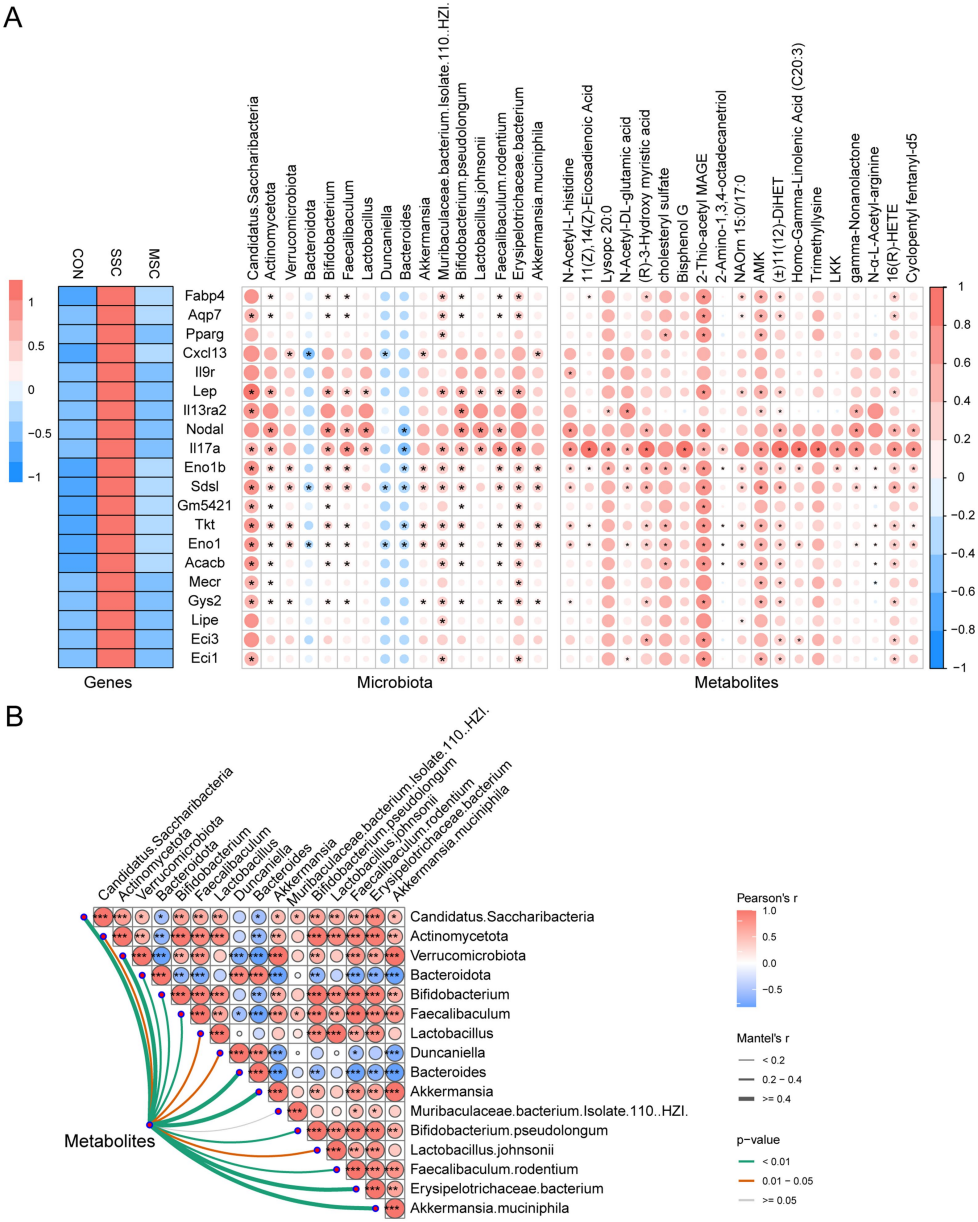
Association analysis of gut microbiota, metabolites, and expression of genes. **(A)** Correlation analysis of differential microbiota (left), metabolites (right) with genes, *p < 0.05. Genes involved in fatty acid metabolic process, AMPK signaling pathway, PPAR signaling pathway, fatty acid biosynthesis, carbon metabolism, and cytokine−cytokine receptor interaction were selected for further analysis. **(B)** Mantel test analysis of differential microbial and metabolites, *p < 0.05.

We performed Spearman correlation analysis to explore the potential relationships among gut microbiota, metabolites, and gene expression in lung tissue. As shown in the heatmap, we screened 20 genes involved in fatty acid metabolic process, AMPK signaling pathway, PPAR signaling pathway, fatty acid biosynthesis, carbon metabolism, and cytokine−cytokine receptor interaction carbon metabolism from DEGs (Figure 8A, left). Spearman correlation analysis showed that the expression levels of DEGs (including Lep, Nodal, Il17a, Eno1b, Tkt, Eno1, Acacb, and Gys2) were significantly correlated with the relative abundance of differential microbial taxa (including *Candidatus Saccharibacteria*, *Actinomycetota*, *Bifidobacterium*, *Faecalibaculum*, *Lactobacillus*, *Bacteroides*, *Bifidobacterium pseudolongum*, *Faecalibaculum rodentium,* and *Erysipelotrichaceae bacterium*) (Figure 8A left & middle), these results suggest that the gut microbiota may influence fibrosis by affecting the expression of genes in lung tissue. Furthermore, the expression levels of the 20 genes and microbial metabolites (2-Thio-acetyl MAGE, NAOrn 15:0/17:0, AMK, (±)11(12)-DiHET and 16(R)-HETE) also showed a significant correlation (Figure 8A left & right). These results indicate that gut microbiota metabolites influence tissue fibrosis in mice. To further explore the potential relationships among the multi-omics data, we conducted a Mantel test, and the results showedthat, with the exception of *Muribaculaceae bacterium Isolate-110 (HZI)*, there was a significant correlation between differential microbiota and microbial metabolites (Figure 8B). Consequently, the mediating effect of gut microbiota on fibrosis-related pathways in SSc mice may be attributed to its potential regulatory influence on microbial metabolites. In summary, MSCs may alleviated pulmonary fibrosis in SSc mice by regulating gut microbiota and microbial metabolites.

## 4 Discussion

SSc is a multisystem autoimmune connective tissue disease characterized by skin and organ fibrosis. While targeted immunosuppressive therapies have shown some efficacy in mitigating fibrosis in SSc patients, these treatments remain suboptimal, underscoring the urgent need for the development of biologic therapies capable of halting or reversing fibrotic processes. Emerging evidence suggests that MSCs may facilitate the repair of tissue fibrosis, although the underlying mechanisms remain inadequately understood. In this study, we used microbial metagenomics, non-targeted metabolomics, and transcriptomics to investigate the potential of MSCs in alleviating pulmonary fibrosis associated with SSc. Our findings indicate that MSCs may exert their effects by modulating fatty acid metabolism, PPAR signaling pathway, and AMPK signaling pathway. Additionally, MSCs appear to have the capacity to restore the dysregulated gut microbiota and microbial metabolites observed in SSc. These insights advance our understanding of the specific mechanisms through which MSCs ameliorate fibrosis and may inform the development of novel therapeutic strategies for the clinical management of SSc.

Fibrosis is the endpoint of many pathologies, characterized by extensive deposition of myofibroblasts and a tough extracellular matrix (ECM) primarily composed of highly cross-linked and stable Type I collagen. Immune abnormalities and abnormal secretion of inflammatory and fibrotic cytokines play an essential role in the pathogenesis of fibrosis in SSc (Fang et al., 2022). Studies have found that MSCs inhibit the fibrotic process of SSc by inhibiting the recruitment of pathogenic macrophages (Gong et al., 2022; Li et al., 2024). In this study, SSc mice were given twice MSCs treatments (5 × 10^5^ hUC-MSCs per mouse/time). MSCs treatment significantly reduced inflammatory cell infiltration, collagen deposition, and the expression of fibrosis-related proteins *α*-SMA and COL1A1 in the lung tissue of SSc mice (Figure 4). Consistent with previous studies, these results suggest that MSCs therapy can effectively alleviate the progression of fibrosis in BLM-induced SSc mouse models. Transcriptomic analysis showed that the pathways most significantly affected by MSCs treatment include changes in the fatty acid metabolic process, fatty acid biosynthesis, PPAR signaling pathway, AMPK signaling pathway, carbon metabolism, and cytokine−cytokine receptor interaction. Fatty acid metabolism plays an important role in the occurrence and development of pulmonary fibrosis. Studies have shown that changes in the expression of genes related to fatty acid metabolism may be a reliable biomarker for predicting the survival of patients with idiopathic pulmonary fibrosis (IPF) (Lyu et al., 2022). Abnormal fatty acid metabolism may lead to the imbalance of monounsaturated fatty acid ratio, thus promoting the occurrence of pulmonary fibrosis (Liu et al., 2024). Fatty acids are responsible for enhancing the expression of the fatty acids receptor CD36, which induces the M2 macrophages by increasing fatty acids uptake and self-reinforcing the profibrotic activation cycle (Parks et al., 2013). M2 macrophages have been shown to promote the onset and progression of tissues fibrosis in SSc. The activation of AMPK and PPAR signaling exerts anti-fibrotic effects by inhibiting myofibroblast activation, collagen synthesis, and TGF-*β*/SMAD signaling (Oruqaj et al., 2015, Liu et al., 2024). However, in certain circumstances, the AMPK and PPAR signaling pathways may promote fibrosis. Studies have found that the AMPK signaling pathway can promote the polarization of M2 macrophages (Kuang et al., 2024). CH25H/25-HC promotes M2 macrophage polarization through the AMPK/STAT6 pathway, thereby aggravating COPD-related pulmonary fibrosis (Li et al., 2025). In addition, the activation of the AMPK signaling pathway may promote the activation of fibroblasts and the generation of extracellular matrix by regulating the dynamics of the cytoskeleton and the nuclear translocation of transcription factors, thereby exacerbating renal fibrosis (Wang et al., 2018). The transcription factor peroxisome proliferator activating receptor (PPAR)-*γ* play an important role in fatty acid metabolism (Christofides et al., 2021). It’s reported that the deficiency of *α*CGRP may promote M2 macrophages polarization by activating the PPARγ signaling pathway, thereby exacerbating pulmonary fibrosis (Lv et al., 2023). Consequently, we hypothesize that the upregulation of gene expression induced by BLM, in conjunction with processes such as fatty acid metabolic process, fatty acid biosynthesis, PPAR signaling pathway, and AMPK signaling pathway, is intricately linked to the progression of SSc fibrosis. MSCs may alleviate lung fibrosis in SSC mouse models by regulating these pathways.

Alterations in the gut microbiota, or microbiota dysbiosis, may be one of the factors that influence the disease state of SSc. Compared with HC, there was no significant change in the α-diversity of the gut microbiota in patients with SSc (Figure 1A). However, previous studies have found a significant increase in the α-diversity of the gut microbiota in patients with SSc (Tan et al., 2022), which may be related to the relatively small sample size in this study. Thus, the sample size needs to be expanded for further analysis and verification. Metagenomics sequencing results showed that compared with CON, the α-diversity index in SSc mice was significantly increased (Figure 6A), indicating that gut microbiota diversity and richness in SSc mice relatively increased. The same results has reported by previous studies (Tang et al., 2021), however, α-diversity returned to a healthy state after MSCs treatment. Consistent with previous results (Tang et al., 2021), our study found that compared with healthy controls, gut microbial community structure and species composition were significantly changed in SSc patients and mice (*p* < 0.05, Figures 1B, 4B; Supplementary Figures S1A,B, S3C–E), indicating that the gut microbial community of SSc patients and mice was in a state of dysbiosis. These results indicate that the mouse model can reflect the gut microbial changes observed in human. After MSCs treatment, the gut microbiota community structure of SSc mice was more similar to that of healthy mice, indicating that MSCs treatment can restore the dysregulated gut microbiota. It has been reported that MSCs therapy can significantly restore microbiota diversity and reverse changes in microbial abundance in mice with colitis, thus restoring gut microbiota balance (Fu et al., 2023; Yang et al., 2022).

In SSc patients, there was a significant reduction in the relative abundances of *Bacteroidota*, *Phocaeicola*, *Eubacterium*, *Phocaeicola coprocola*, and *Parabacteroides* (Figures 1C–E; Supplementary Figures S1C,D). Studies have shown that *Bacteroidota*, *Phocaeicola*, *Eubacterium*, *Phocaeicola coprocola*, *Faecalibacterium prausnitzii*, and other microbial groups are important probiotics for humans. It is essential for maintaining gut homeostasis and anti-inflammation by producing short-chain fatty acids (SCFAs), such as butyric acid (Mann et al., 2024; Volkmann et al., 2017). Studies have shown that *Parabacteroides* can play an immunomodulatory role by regulating interleukin-10 (IL-10) levels and Treg cells and producing SCFAs (Zhang et al., 2022; Zeng et al., 2019). Therefore, we speculate that the disturbed gut microbiota may be related to SSc inflammation and fibrosis. In SSc mouse models, the relative abundances of *Bacteroidota* and *Bacteroidesin* were significantly decreased, while *Actinomycetota*, *Bifidobacterium*, *Lactobacillus*, and *Akkermansia muciniphila* were observed to be more abundant in SSc mice, which is consistent with previous studies (Tan et al., 2022; Volkmann et al., 2017; Natalello et al., 2020; Volkmann et al., 2016; Patrone et al., 2017). Compared with the healthy controls, the relative abundance of *Bacteroidota* was significantly decreased in both SSc patients and mice. *Bacteroides* is believed to play a protective role for the host by mitigating mucosal inflammation and inhibiting the colonization of pathogenic species (Round and Mazmanian, 2009). Specifically, *Bacteroides fragilis* can reduce the level of oxidative stress and inflammatory response by reducing serum lipopolysaccharide (LPS) levels and increasing 1,5-anhydroglucitol (1,5-AG) levels to alleviate renal fibrosis (Zhou et al., 2022). The increased relative abundance of *Actinomycetota* in SSc was found to be associated with gastrointestinal involvement (Patrone et al., 2017), suggesting a potential pathogenic role of this genus in SSc. Gastrointestinal dysfunction adversely affects nearly all SSc patients, and the severity of gastrointestinal disease in SSc correlates with high mortality (McMahan et al., 2023). Studies have shown that *Akkermansia muciniphila* can promote the integrity of the intestinal barrier by breaking down the mucus in the intestine, thus playing a protective role in a variety of metabolic and gastrointestinal diseases (Bae et al., 2022). In some cases, *A. muciniphila* may exacerbate intestinal inflammation. Studies have found that *A. muciniphila* may lead to excessive degradation of intestinal mucus in some cases, thus exacerbating inflammation (Ring et al., 2019). Therefore, *Bacteroidota*, *Actinomycetota*, and *Akkermansia muciniphila* may affect the occurrence and development of SSc fibrosis by altering the intestinal barrier function. Further studies on the relationship between gut microbiota and SSc and its effect of immune regulation are needed in the future.

In addition to the gut microbiota, microbial metabolites play a crucial role in maintaining immune homeostasis and modulating fibrotic responses. Therefore, exploring the effect of gut microbial metabolites on tissue fibrosis in SSc is of great significance. Consistent with previous findings (Volkmann et al., 2017), our study found an altered pattern of gut microbial metabolites in both SSc patients and mice (Figures 2A,B). In human samples, the DEMs were mainly involved in the protein digestion and absorption, aminoacyl-tRNA biosynthesis, sphingolipid metabolism, and biosynthesis of unsaturated fatty acid. Notably, alterations in sphingolipid metabolism are associated with SSc and have been demonstrated to promote fibrosis in dermal fibroblast cultures (Samuel et al., 2012; Tan et al., 2022). As an important dietary nutrient, unsaturated fatty acids are closely related to various diseases. Studies have shown that unsaturated fatty acids may influence the development of pulmonary fibrosis through a variety of mechanisms. For instance, one study found that the imbalance of monounsaturated fatty acid ratio was significantly associated with pulmonary fibrosis through metabolomics and mendelian randomization analysis, suggesting that unsaturated fatty acid may play an important role in the occurrence of pulmonary fibrosis (Liu et al., 2024). In addition, unsaturated fatty acids have been shown to improve blood lipid levels, reduce low-density lipoprotein cholesterol (LDL-C), and raise high-density lipoprotein cholesterol, thereby potentially reducing the risk of cardiovascular disease (Mente et al., 2017).

In mice samples, the DEMs were predominantly associated with pathways such as nicotinate and nicotinamide metabolism, pantothenate and CoA biosynthesis, neuroactive ligand−receptor interaction, and inflammatory mediator regulation of TRP channels (Figures 7L,M). These findings suggest that MSCs treatment may alleviate metabolic disorders by altering the gut microbial metabolic processes in SSc. It’s reported that nicotinate and nicotinamide metabolism may play a role in inflammation (Ma et al., 2016; Gong et al., 2020). The involvement of nicotinate and nicotinamide metabolism in the inflammatory response may be associated with various metabolic enzymes and secondary metabolites generated during these processes. Notably, nicotinate phosphoribosyltransferase serves as a critical inflammatory mediator by binding to Toll-like receptor 4, thereby triggering the activation of inflammasomes and the NF-κB pathway. This activation subsequently results in the secretion of inflammatory mediators, including IL-1*β*, IL-8, and TNF-α (Managò et al., 2019). Our study demonstrated a significant reduction in trimethyllysine and cholesteryl sulfate levels in SSc mice following MSCs treatment. Trimethyllysine is a precursor to the gut microbiome derived metabolite trimethylamine N-Oxide (TMAO). Studies have found that the concentration of TMAO is increased in the plasma of patients with SSc organ involvement, suggesting a potential link between dysbiosis and organ involvement in the pathogenesis of the disease (Stec et al., 2023). Additionally, studies have shown that fibroblasts, when stimulated by cholesteryl sulfate, increase the synthesis of ECM components, such as collagen and fibronectin. This phenomenon is related to the activation of the transforming growth factor-β (TGF-β) signaling pathway by cholesteryl sulfate. Combined with our results, we concluded that MSCs may influence SSc pulmonary fibrosis by regulating gut microbial metabolites.

Our studies reveled that MSCs therapy can improve fibrosis and restore the disordered gut microbial composition in SSc mice. Correlation analysis results showed that DEGs were significantly correlated with differential microbial taxa and metabolites (Figure 8). This suggests that gut microbiota and their metabolites influence the progression of pulmonary fibrosis. Collectively, we propose that MSCs may alleviated pulmonary fibrosis in SSc by modulating the gut microbiota. Studies have shown that the gut microbiota and their metabolites play a significant role in the occurrence and development of pulmonary fibrosis. Alterations in the gut microbiota can either exacerbate or alleviate pulmonary fibrosis by influencing the pulmonary immune response and inflammatory processes (Ruan et al., 2024; Gurczynski et al., 2023). Dysregulation of the gut microbiota can exacerbate pulmonary fibrosis by enhancing the IL-17A signaling pathway (Yamada et al., 2023). Gut microbiota dysbiosis is associated with the severity of pulmonary fibrosis, and modulating the gut microbiota can alleviate symptoms of the disease (Chioma et al., 2022). The metabolites of the gut microbiota, such as SCFAs, bile acids, and tryptophan derivatives, can affect the progression of pulmonary fibrosis by regulating immune responses and fibrosis signaling pathways. For instance, SCFAs can inhibit inflammatory responses by binding to G protein-coupled receptors (such as GPR41, GPR43, and GPR109A), thereby slowing down the progression of pulmonary fibrosis to a certain extent (Sivaprakasam et al., 2016). Tryptophan metabolites promote the progression of pulmonary fibrosis by activating the mTOR/S6 pathway, while modulation of certain microbial communities may slow down fibrosis by inhibiting these pathways (Li et al., 2024). Therefore, in-depth research on the impact of MSCs on the gut microbiota is expected to provide new possibilities for the prevention, diagnosis and treatment of SSc pulmonary fibrosis.

While our study investigated a potential mechanism through which MSCs may alleviate pulmonary fibrosis in SSc, certain limitations should be acknowledged. Firstly, we have not yet established a direct causal relationship between gut microbiota dysbiosis and the onset of pulmonary fibrosis in SSc. To address this gap, future research will involve fecal microbiota transplantation experiments to elucidate the specific role of gut microbiota in SSc. Secondly, our study lacks validation studies; thus, we intend to employ an *in vitro* cell culture system to verify and enhance our findings through a multidimensional approach.

## 5 Conclusion

This study elucidates the sffects of MSCs therapy on gut microbiota within the context of SSc, thereby advancing our understanding of the mechanisms underlying onset and progression of SSc. Our findings suggest that MSCs can attenuate SSc-associated pulmonary fibrosis by modulating fatty acid metabolism, PPAR signaling pathway, and AMPK signaling pathway. Additionally, MSCs demonstrate the potential to restore the dysregulated gut microbiota and microbial metabolites characteristic of SSc. The study further reveals that the altered gut microbiota in SSc affects signaling pathways associated with pulmonary fibrosis. These results underscore the importance of future research into the specific role of gut microbiota in fibrosis, which could enhance our comprehension of SSc pathogenesis and potentially identify novel therapeutic targets for biological interventions in SSc.

## Data Availability

The data presented in this study are publicly available. The data can be found at: https://www.ncbi.nlm.nih.gov/, accession PRJN1124507.

## Footnotes

1
https://github.com/cjfields/readfq

2
https://github.com/bbuchfink/diamond/

3
https://www.ncbi.nlm.nih.gov/

4
https://en.wikipedia.org/wiki/Lowest_common_ancestor

5
www.ncbi.nlm.nih.gov/sra
