# Supplementary material for: Mesenchymal stem cells alleviate pulmonary fibrosis and gut microbiota dysbiosis in systemic sclerosis
Source: Front Microbiol. 2025 Nov 26;16:1635809. doi: 10.3389/fmicb.2025.1635809 (PMC12693401; doi:10.3389/fmicb.2025.1635809)
Supplement: Supplementary file 1 [file Supplementary_file_1.docx]

Supplementary Material

Mesenchymal stem cells alleviate pulmonary fibrosis and gut microbiota dysbiosis in systemic sclerosis

**Biao Ni^1,2†^, Yufang Gong^3†^,** **Bin Li^1†^,** **Lijie Qiu^1†^, Kang He^4^, J****intao Guo^2^, Hongkun Fang^2^, Mingjie Gao^2^, Min Chen^2^, Cuie Wei^3^, Weice Sun^5^, Bin Liu^6^,** **Ming Li^3*^,** **Shaoqiang Wang^2*^,** **Lina Xu^1*^**

^1^Department of Pulmonary and Critical Care Medicine, Center of Respiratory Medicine, Weifang People's Hospital, Shandong Second Medical University, Weifang，261041, Shandong Province, China.

^2^Department of Scientific Research Management, Weifang People's Hospital, Shandong Second Medical University, Weifang, 261041, Shandong Province, China.

^3^Department of Rheumatology, Weifang People's Hospital, Shandong Second Medical University, Weifang, 261041, Shandong Province, China.

^4^Department of Clinical Medicine of Shandong Second Medical University, Weifang, 261000, Shandong Province, China

^5^Vascular Surgery, Weifang Hospital of Traditional Chinese Medicine, Weifang, 261000, Shandong Province, China

^6^Weifang People's Hospital, Shandong Second Medical University, Weifang, 261041, Shandong Province, China.

†: These authors contributed equally to this work.

*** Correspondence:**
Lina Xu (xulina0201@126.com), Shaoqiang Wang (rmyywsq227@sdsmu.edu.cn), Ming Li (lalwlm@aliyun.com).

The following Supporting Information is available for this article:


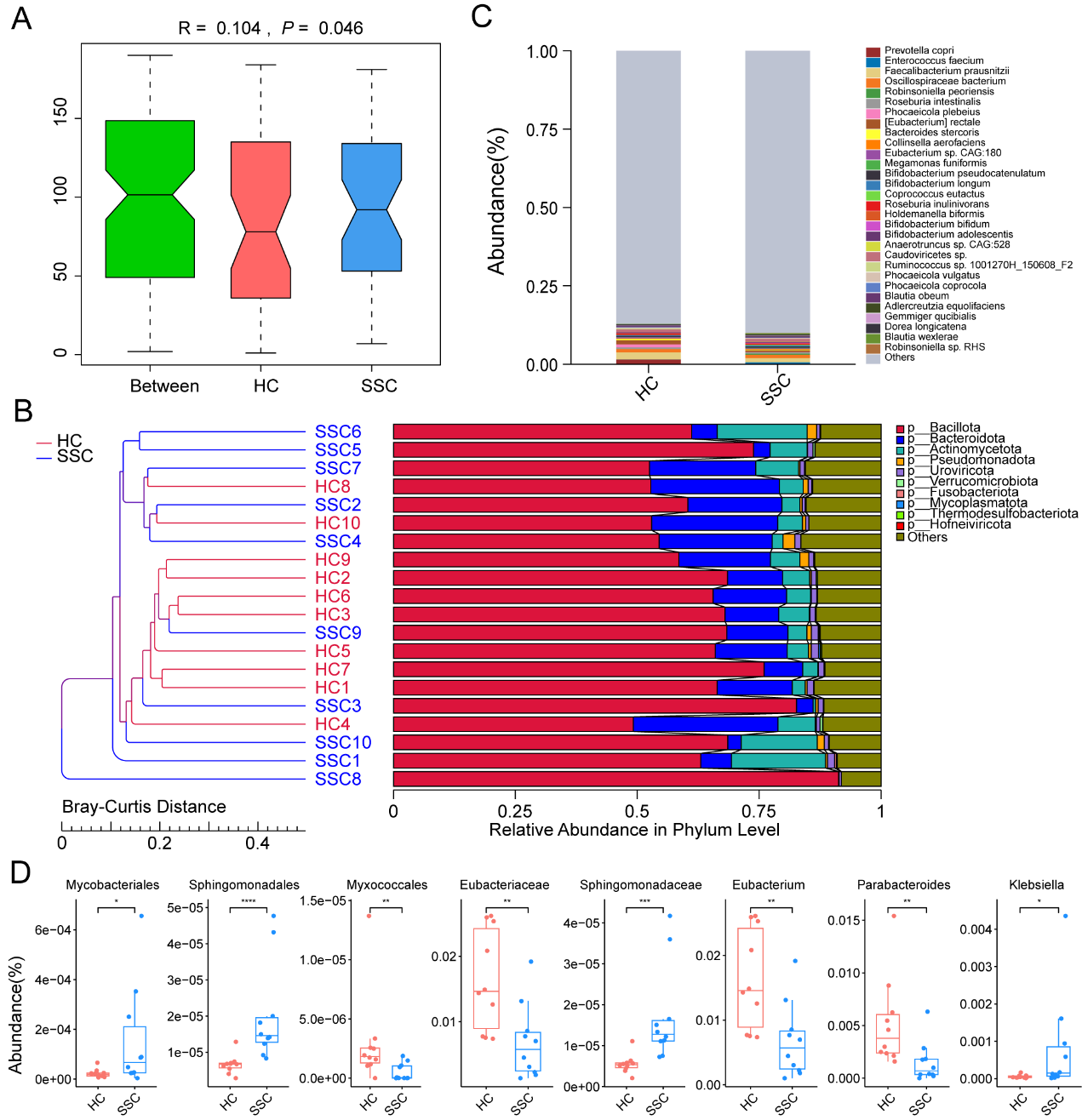


**Figure S1.** **Community composition of gut microbiota.** (A) ANOSIM analysis of gut microbiota between the HC and SSC groups. (B) Clustering analysis of gut microbiota based on Bray-Curtis distance at phylum level. (C) Relative abundance of the 30 most abundant genus of gut microbiota. (D) Relative abundance of the significantly altered microbial taxa from the two groups.


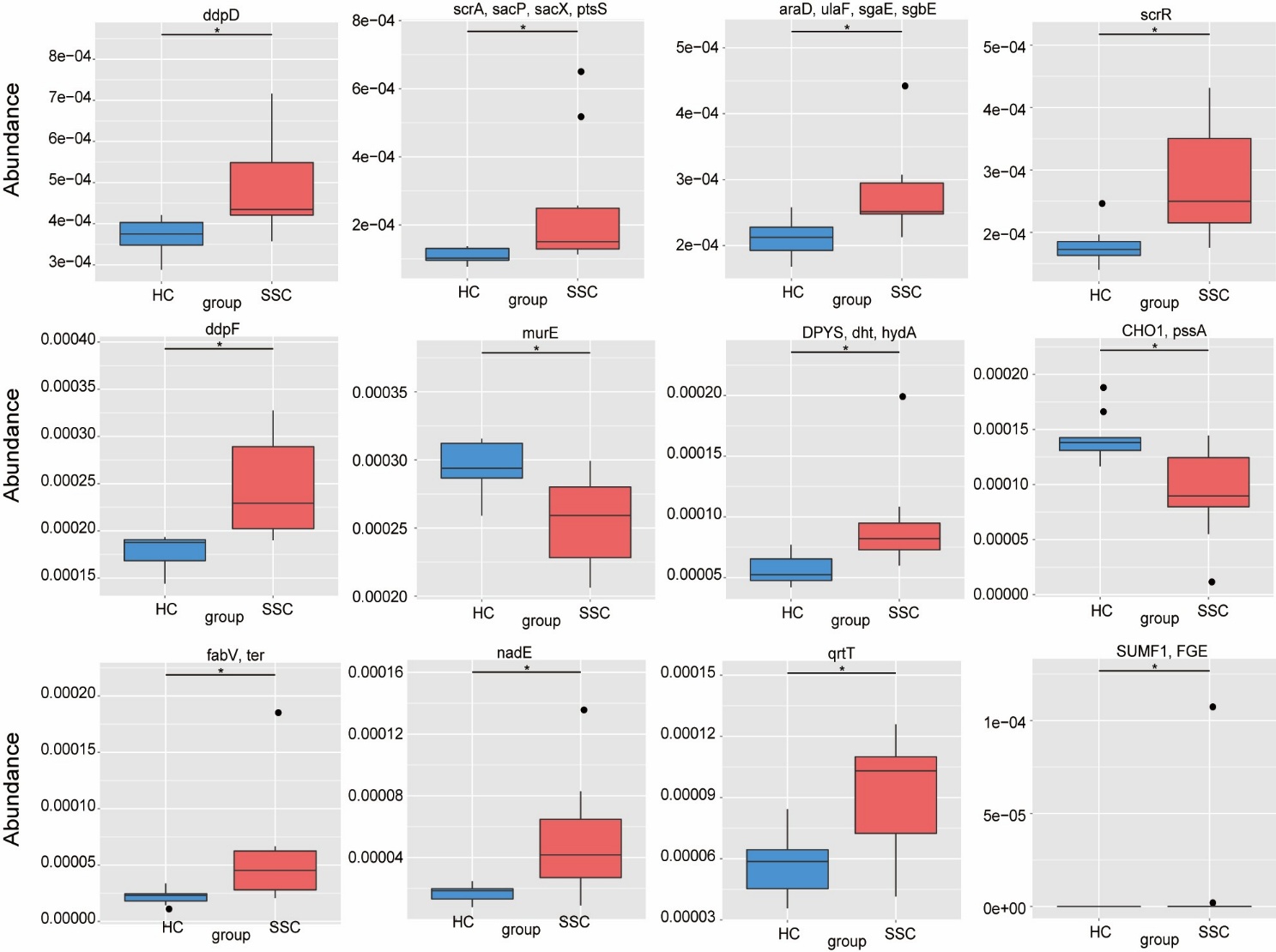


**Figure S2.** Differential analysis of microbial function.


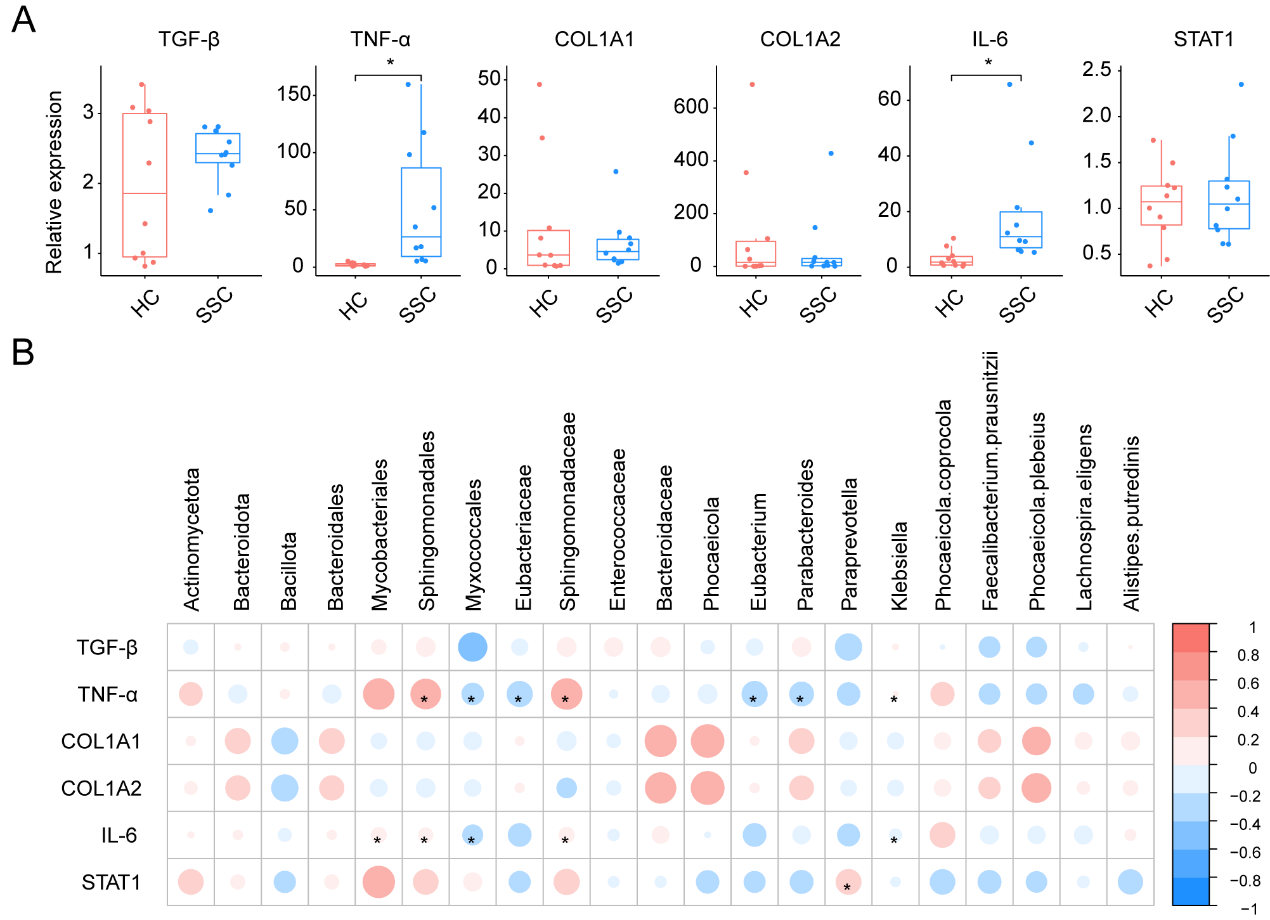


**Figure S3.** **Inflammation was increased in SSc patients and correlated with gut microbiota.** (A) Analysis of mRNA expression levels of inflammatory and fibrotic factors, **P*<0.05. (B) Spearman correlation analysis between differential microbes and mRNA expression of inflammatory and fibrotic factors, **P*<0.05.


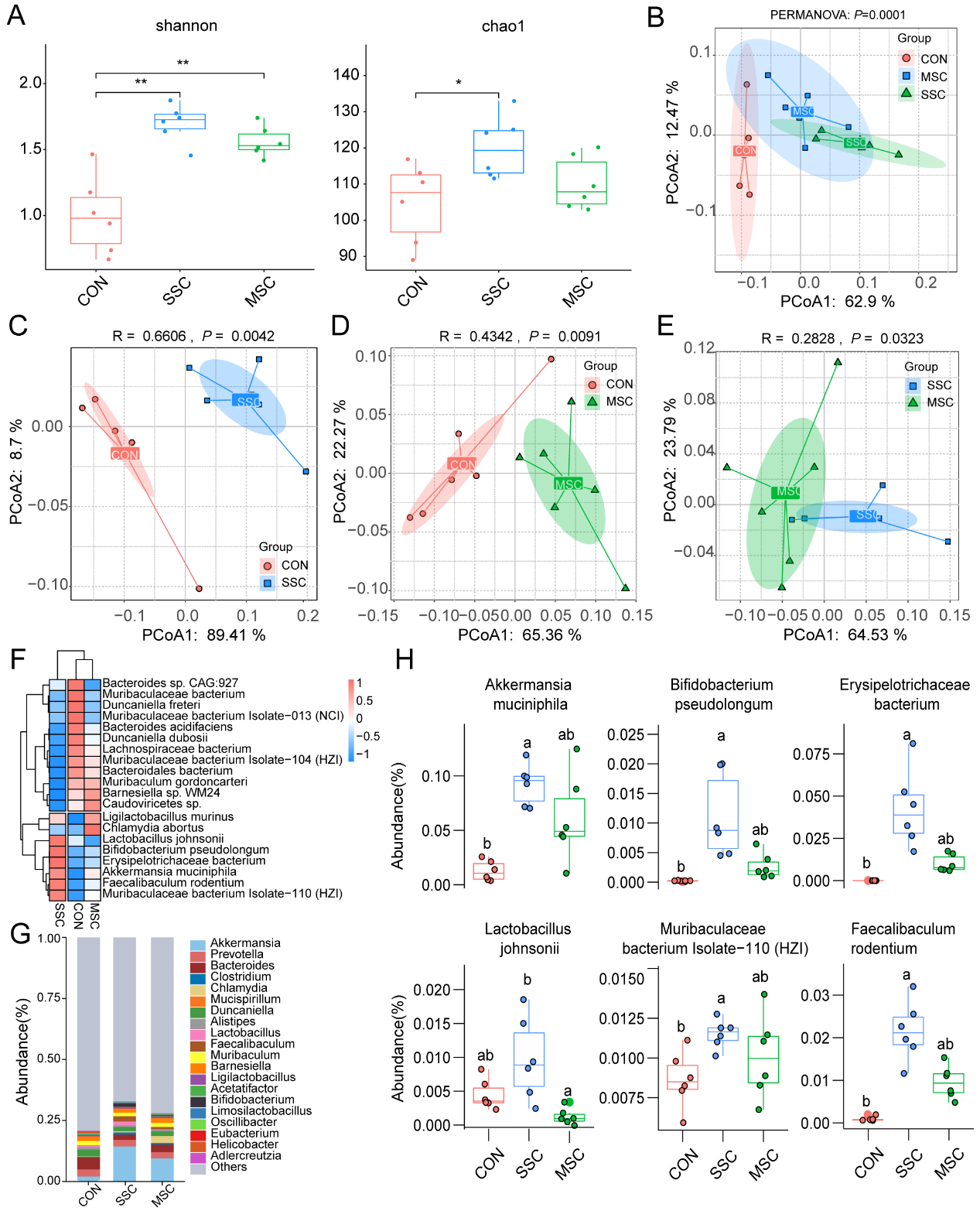


**Figure S4. MSCs improved the gut microbiota dysbiosis in SSc mice** (A) Alpha diversity of gut microbiota in mice. (B) Principal coordinate analysis (PCoA) of microbiota community based on Bray-Curtis distance at species level. (C-E) PCoA of microbiota community based on Bray-Curtis distance at phylum level. (F) Clustering heatmap of gut microbiota at the species level. (G) Relative abundance of gut microbiota at the species level. (H) Relative abundance of the significantly altered microbial taxa at the species level from the three groups.

Table S1. Primers used for Quantitative real-time PCR (qPCR).

| Primer | Sequences ( 5’-3’) |
| --- | --- |
| Human-GAPDH-F | GGAGCGAGATCCCTCCAAAAT |
| Human-GAPDH-R | GGCTGTTGTCATACTTCTCATGG |
| Human-TGF-β-F | CCTGGCGATACCTCAGCAACC |
| Human-TGF-β-R | CCTCCACGGCTCAACCACTG |
| Human-TNF-α-F | GGCGTGGAGCTGAGAGATAACC |
| Human-TNF-α-R | CGGCTGATGGTGTGGGTGAG |
| Human-COL1A1-F | CGGTGAACCTGGTGCTCCTG |
| Human-COL1A1-R | GCTCCTCGCTTTCCTTCCTCTC |
| Human-COL1A2-F | AGTTGGACCTGCTGGCATTCG |
| Human-COL1A2-R | ATAACCACCACCGCTTACACCTG |
| Human-IL-6-F | TGGTGTTGCCTGCTGCCTTC |
| Human-IL-6-R | GCTGAGATGCCGTCGAGGATG |
| Human-STAT1-F | CGACCTCTCTGCCCGTTGTG |
| Human-STAT1-R | TCCGCCACCAGCATGTTGTAC |
